# Pachymic Acid Inhibits Growth and Induces Apoptosis of Pancreatic Cancer In Vitro and In Vivo by Targeting ER Stress

**DOI:** 10.1371/journal.pone.0122270

**Published:** 2015-04-27

**Authors:** Shujie Cheng, Kristen Swanson, Isaac Eliaz, Jeanette N. McClintick, George E. Sandusky, Daniel Sliva

**Affiliations:** 1 Cancer Research Laboratory, Methodist Research Institute, Indiana University Health, Indianapolis, Indiana, United States of America; 2 Amitabha Medical Clinic and Healing Center, Santa Rosa, California, United States of America; 3 Departments of Biochemistry and Molecular Biology, School of Medicine, Indiana University, Indianapolis, Indiana, United States of America; 4 Departments of Pathology, School of Medicine, Indiana University, Indianapolis, Indiana, United States of America; 5 Departments of Medicine, School of Medicine, Indiana University, Indianapolis, Indiana, United States of America; 6 DSTest Laboratories, Purdue Research Park, Indianapolis, Indiana, United States of America; Complutense University, SPAIN

## Abstract

Pachymic acid (PA) is a purified triterpene extracted from medicinal fungus *Poria cocos*. In this paper, we investigated the anticancer effect of PA on human chemotherapy resistant pancreatic cancer. PA triggered apoptosis in gemcitabine-resistant pancreatic cancer cells PANC-1 and MIA PaCa-2. Comparative gene expression array analysis demonstrated that endoplasmic reticulum (ER) stress was induced by PA through activation of heat shock response and unfolded protein response related genes. Induced ER stress was confirmed by increasing expression of XBP-1s, ATF4, Hsp70, CHOP and phospho-eIF2α. Moreover, ER stress inhibitor tauroursodeoxycholic acid (TUDCA) blocked PA induced apoptosis. In addition, 25 mg kg^-1^ of PA significantly suppressed MIA PaCa-2 tumor growth *in vivo* without toxicity, which correlated with induction of apoptosis and expression of ER stress related proteins in tumor tissues. Taken together, growth inhibition and induction of apoptosis by PA in gemcitabine-resistant pancreatic cancer cells were associated with ER stress activation both *in vitro* and *in vivo*. PA may be potentially exploited for the use in treatment of chemotherapy resistant pancreatic cancer.

## Introduction

The high mortality rates of pancreatic cancer, one of the most lethal cancers worldwide, are the result of inadequate tools for early diagnosis and few therapeutic options [1]. Gemcitabine, a cytotoxic nucleoside analogue, is the current clinical standard of care for advanced pancreatic cancer but has a response rate of less than 20% [2]. A potential novel strategy for highly treatment-resistant cancers is the induction of an organelle-related stress response, such as endoplasmic reticulum (ER), nuclear and mitochondrial stress response in cancer cells [3]. Considering that pancreatic cancer cells have a prominent ER [4, 5], this organelle may represent an attractive therapeutic target.

ER is the principal site of protein synthesis, folding and modification [6]. Several physiological conditions such as oxidative stress, glucose deprivation and infection can lead to disturbances in the ER [7]. Under these conditions, protein folding is disrupted and unfolded or misfolded proteins accumulate in the ER lumen, which lead to ER stress [8, 9]. To overcome the effects of ER stress, a series of adaptive and protective strategies have evolved, including two highly conserved systems—the heat shock response (HSR) and the unfolded protein response (UPR) [10]. Both systems act as quality control processes to facilitate protein folding and suppress protein synthesis [10, 11]. HSR involves stimulation of factors and pathways, such as inducing the synthesis of molecular chaperones (e.g., Hsp70), to attenuate the burden of misfolded proteins [10, 12]. UPR is initiated by three ER transmembrane receptors: activating transcription factor-6 (ATF6), inositol-requiring enzyme 1 (IRE1) and PKR-like ER kinase (PERK) [13]. Among of them, PERK appears to play the major role in the face of mild ER stress. The activated PERK kinase phosphorylates the alpha subunit of the eukaryotic initiation factor 2 (eIF2α), which results in the first step in the UPR: decreased translation initiation and protein synthesis [14, 15].

As a pro-survival and protective response, UPR aims at reducing the backlog of unfolded or misfolded proteins to restore normal ER function [16]. However, if the stress cannot be resolved, signaling through ATF6, IRE1 and PERK trigger pro-apoptotic response through activation of downstream molecules, such as transcription factor C/EBP homologous protein (CHOP), which further induce apoptosis during prolonged ER stress [11].

A number of pharmacological agents that directly or indirectly induce ER stress have been shown to trigger apoptotic cell death of pancreatic cancer. Edelfosine, an alkyl-lysophospholipid analog, induces ER stress and apoptosis *in vitro* and *in vivo* through its accumulation in the ER [17]. 1, 1-bis (3'-indoly)-1-(p-substituted phenyl) methanes activate ER stress-mediated apoptosis in pancreatic cancer in a structure-dependent manner [18]. 3, 3'-diindolylmethane and its derivatives induce apoptosis through ER stress-dependent upregulation of death receptor in pancreatic cancer cells [19]. Bortezomib, a potent and selective inhibitor of proteasome, sensitizes pancreatic cancer cells to ER stress-induced apoptosis and strongly enhances the antitumor activity of cisplatin [20].

*Poria cocos* is a medicinal fungus in the *Polyporaceae* family that grows in pine trees and its sclerotium is widely used in traditional Asian medicine for its sedative, diuretic, digestive and tonic effects [21–23]. Pachymic acid (PA), a triterpenoid from *P*. *cocos*, has been reported to possess anticancer properties in different types of cancer cells [24–28]. However, the effect of PA against pancreatic cancer remains to be evaluated and the mechanism is not clear. Previous work in our laboratory has shown that PA suppresses growth and invasiveness of pancreatic cancer cells through downregulation of MMP-7 [29]. In the present study, using various *in vitro* and *in vivo* experimental approaches including comparative gene expression array analysis as a discovery tool, we report for the first time that PA, behaves as an effective anticancer agent, inhibits growth and induces apoptosis of chemotherapy resistant pancreatic cancer cells by targeting ER stress.

## Materials and Methods

### Cell culture and reagents

The human pancreatic cancer cell lines PANC-1 and MIA PaCa-2 were obtained from ATCC (Manassas, VA). PANC-1 cells were maintained in Dulbecco's modified Eagle's medium containing penicillin (50 U ml^-1^), streptomycin (50 U ml^-1^) and 10% fetal bovine serum (FBS). MIA PaCa-2 cells were maintained in Dulbecco's modified Eagle's medium containing penicillin (50 U ml^-1^), streptomycin (50 U ml^-1^), 10% FBS and 2.5% horse serum (HS). Media came from ATCC. Supplements, FBS and HS were obtained from Gibco BRL (Grand Island, NY). DMSO was purchased from Sigma (St. Louis, MO). All other chemicals and reagents were of analytical grade. Anti-cleaved PARP antibody was obtained from BD Biosciences (San Jose, CA and anti-β-actin antibody was obtained from Santa Cruz Biotechnology (Santa Cruz, CA). Anti-CHOP, anti-phospho-eIF2α and anti-eIF2α antibodies were obtained from Cell Signaling Technology (Beverly, MA). Anti-Hsp70 was obtained from Enzo Life Sciences (Farmingdale, NY). Anti-XBP-1s from BioLegend (San Diego, CA), anti-ATF6 and anti-ATF4 from Abcam (Cambridge, MA) were kindly provided by Dr. Michael A.J. Zieger from Cryobiology Laboratory, Methodist Research Institute, Indiana University Health. Tauroursodeoxycholic acid (TUDCA) was purchased from Calbiochem (Darmstadt, Germany). PA was purchased from Shinning-biotech (Chengdu, Sichuan, China) and dissolved in DMSO at a concentration of 50 mM then stored at -20°C.

### Determination of apoptosis

Apoptosis induction in PA-treated cells was assessed by quantitation of cytoplasmic histone-associated DNA fragments and western blotting for PARP cleavage (c-PARP). The Cell Death Detection ELISA^PLUS^ Kit (Roche, Indianapolis, IN) was used to detect the amount of cytoplasmic histone-associated DNA fragments according to the manufacturer’s instructions and expressed relative to vehicle-treated cells (set equal to 1).

### DNA microarray analysis

MIA PaCa-2 cells were treated with PA (0 and 30 μM) for 24 h in quadruplicates. Total-RNA from each sample was isolated with RNeasy Mini Kit (Qiagen, Valencia, CA) according to the manufacturer’s protocol. The integrity of the isolated RNA was checked on an Agilent Bioanalyzer (Agilent Technologies, Palo Alto, CA). The Center for Medical Genomics at Indiana University School of Medicine received total RNA and carried out all the steps of processing the RNA, hybridization to the Affymetrix Human PrimeView GeneChips, washing, scanning and initial analysis. The 3’IVT express kit from Affymetrix (Affymetrix, Santa Clara, CA) was used to label the samples. The CEL files were scanned for defects and none were found, The CEL files were imported into Partek Genomics Suite for analysis (Partek, Inc., St. Louis, Mo) RMA (Robust Multi-Array Average) signals were generated for the core probe sets using the RMA background correction, quantile normalization and summarization by Median Polish [30]. Summarized signals for each probe set were log2 transformed. These log-transformed signals were used for Principal Components Analysis, Hierarchical Clustering and Signal Histograms to determine if there were any outlier arrays, no outliers were detected. Untransformed RMA signals were used for fold change calculations. Data was analyzed by 2-way ANOVA using cell line and treatment as factors plus the interaction of cell line and treatment. The q-value program from Storey and Tibshirani was used to calculate false discovery rates.

### Western blot analysis

PANC-1, MIA PaCa-2 cells were treated with PA (0–30 μM) for 10 min, 30 min or 24 h, respectively. Whole protein extracts isolated from cells and tumor tissues were prepared and western blot analysis with anti-cleaved PARP, anti-XBP-1s, anti-ATF6, anti-ATF4, anti-Hsp70, anti-CHOP, anti-phospho-eIF2α or anti-eIF2α antibodies were performed as previously described [31]. Western blots were quantified with HP-Scanjet 550c and analyzed by UN-SCAN-IT software (Silk Scientific, Orem, UT). Three independent experiments were done for all the immunoblot studies and quantitative data composed of all the experiments with statistical analysis were added below the representative blot image.

### Human pancreatic tumor xenograft experiment

Animal experiments were conducted strictly in accordance with the protocol approved by the Animal Research Committee at the Methodist Hospital (protocol no. 2011–07). MIA PaCa-2 cells (3x10^6^) in 100 μl DPBS were implanted subcutaneously in the right flank on the ventral side of the 6 weeks old female nude mice (Harlan, Indianapolis, IN, USA). When tumors became palpable after 1–3 weeks (40–60 mm^3^), animals were randomly assigned into three groups (n = 15) and received intraperitoneal (i.p.) injection 3 times per week of vehicle or PA (25 mg kg^-1^ and 50 mg kg^-1^) of body weight for additional 5 weeks. Tumor sizes were measured 3 times per week with microcalipers and the tumor volumes were calculated with the standard formula: tumor volume (mm^3^) = L x W^2^ x 0.5, where L is the length and W is the width of the tumor. At the end of the experiment (Day 35), the mice were euthanized by CO_2_ inhalation. The tumors were harvested and fixed in 10% neutral-buffered formalin at 4°C for 24 h or snap frozen and stored separately in liquid nitrogen.

### Apoptosis measurement in tumor xenograft

All formalin-fixed tumors were embedded in paraffin within 48 h and five-micrometer sections were stained with hematoxylin and eosin (H&E). The slides were viewed under a Leica microscope (Leica Instruments, Melville, NY) and apoptosis in the viable tumor cell area was quantified by counting apoptotic bodies in four 20 x fields of view for n = 6–12 tumors per group by 2 independent observers.

### Toxicology studies *in vivo*

Toxicity of PA was evaluated in the 6 weeks old female nude mice (Harlan, Indianapolis, IN). The mice were acclimatized for 1 week and treated with PA (0, 25 or 50 mg kg^-1^ of body weight, n = 15 per group) by i.p. 3 times per week for additional 5 weeks. The body weight was evaluated 3 times per week. At the end of the experiment, animals were euthanized by CO_2_ inhalation. Blood was collected and a gross pathology examination performed. Liver, spleen, kidney, lung and heart were harvested, fixed in 10% neutral-buffered formalin at 4°C for 24 h followed tissue processing overnight and then embedded in paraffin. Five-micrometer sections were stained with H&E. The levels of alkaline phosphatase (ALP), alanine aminotransferase (ALT), aspartate aminotransferase (AST), albumin and total protein were determined at the IU Health Pathology Laboratory (Indianapolis, IN).

### Quantitative RT-PCR

The quantitative real-time polymerase chain reaction (qRT-PCR) was performed using ABI PRISM 7900HT Fast Real-Time PCR System (Applied Biosystems, Foster City, CA) according to the manufacturer's instructions. Total RNA was isolated from tumors with RNeasy Mini Kit (Qiagen, Valencia, CA). RNA quality was monitored and quantified using the Qubit 2.0 Fluorometer (Invitrogen, Carlsbad, CA). The RNA samples were reverse transcribed into cDNA using High Capacity cDNA Reverse Transcription Kit (Applied Biosystems, Foster City, CA). The cDNA (100 ng per sample) was subjected to qPCR analysis in triplicate. Analysis of the relative quantity gene expression (RQ) of *HSPA6* and *CHOP* was normalized by *ACTB* expression and was performed using the 2^-ΔΔCt^ method [32].

### Statistical analysis

All the statistical analysis was performed using SigmaPlot 11.2.0 (Systat Software Inc., San Jose, CA). Data were presented as mean ± SD. Statistical comparisons between two groups of data were carried out using Student's t-test and comparisons between many groups of data were carried out using ANOVA with the significance level adjusted using the repeated t-tests with Bonferroni correction.

## Results

### PA induces apoptosis in chemoresistant pancreatic cancer cells

The survival outcome of pancreatic cancer is associated with chemotherapy resistant to current chemotherapy regimens [33]. Gemcitabine results in a tumor response rate of 12% and offers a median survival time of 5 months, which means that the best current treatment offers very modest benefits [34]. Altered cell apoptosis is considered to be an important mechanism in pancreatic cancer chemotherapy resistant and is an attractive research area [33].

**Fig 1:**
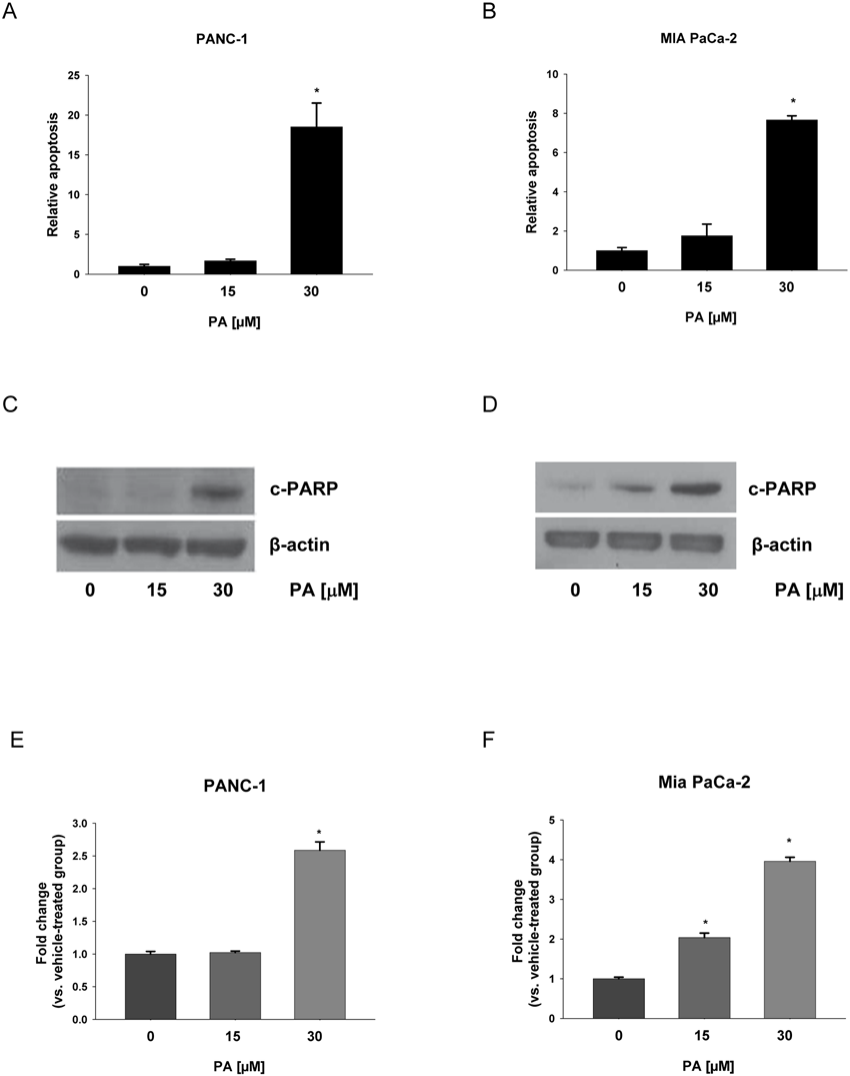
PA induces apoptosis in chemotherapy resistant pancreatic cancer cells. (A) PANC-1 and (B) MIA PaCa-2 cells were treated with PA (0–30 μM) for 24 h. Apoptosis was detected by Cell Death Detection ELISA and expressed relative to vehicle-treated cells (set equal to 1). Data were compared by ANOVA with Bonferroni correction for the significant level. Here, *p≤0.025 was considered significant for the individual t-tests. Apoptosis was confirmed in C) PANC-1 and D) MIA PaCa-2 cells treated with same concentrations of PA for 24 h. Representative blots show expression of PARP cleavage (c-PARP) and β-actin was used as loading control. Three independent experiments were done for the western blot studies and quantitative data composed of all the experiments in E) PANC-1 and F) MIA PaCa-2 cells with statistical analysis were shown below the representative blot image. *P<0.050 was considered to be significant when compared to control (n = 3) by Student t-test. The graphical data represent mean +/- SD.

It was reported that pancreatic cancer cell lines PANC-1 and MIA PaCa-2 were resistant to gemcitabine treatment [35–37]. In addition, we previously demonstrated that PA inhibits proliferation of PANC-1 (IC50-24 h = 23.49 μM) and MIA PaCa-2 (IC50-24 h = 26.61 μM) cells, respectively [29]. The apoptosis inducting effects of PA in gemcitabine-resistant PANC-1 and MIA PaCa-2 were determined by the induction of nuclear DNA fragmentation using ELISA [38]. Treatment of PANC-1 or MIA PaCa-2 cells with different concentrations of PA for 24 h resulted in an increase in apoptosis (Fig 1A and 1B). PA-induced apoptosis was nearly 19 fold in PANC-1 cells and 8 fold in MIA PaCa-2 cells comparing with their respective controls (Fig 1A and 1B).

Apoptosis inducing effects of PA were further confirmed by increased expression of cleaved PARP (c-PARP). PARP is a 116-kDa nuclear poly (ADP-ribose) polymerase, which is involved in DNA repair in response to environmental stress and cleavage of PARP facilitates cell disassembly [39, 40]. PANC-1 and MIA PaCa-2 cells were exposed to different concentrations of PA for 24 h and the c-PARP was detected by western blot analysis. As expected, PARP cleavage was clearly observed in PA treated cells by the presence of an 89-kDa fragment (Fig 1C and 1D). Quantification results showed approximately 3 fold increases in the expression of PARP cleavage in PANC-1 cells and 4 fold increases in MIA PaCa-2 cells by PA treatment comparing with their vehicle-treated controls (Fig 1C and 1D).

### PA upregulates expression of ER stress related genes

In order to gain further mechanistic insight into the molecular events underlying gemcitabine-resistant pancreatic cancer cell-directed apoptosis of PA, the Human Prime View Array was used. This gene expression array was designed using the 3’ end of the transcript. Sequences used in the array were selected from the RefSeq version 36, UniGene database 219, and full-length human mRNAs from GenBank. There are 36,000 transcripts (genes plus some variants) interrogated on the array. All data have been deposited in NCBI's Gene Expression Omnibus and are accessible through GEO Series accession number GSE64111.

In the present investigation, 191 genes were significantly upregulated in MIA PaCa-2 cells after 30 μM PA treatment (fold change threshold = 1.50; ANOVA, Storey-Tibshirani false discovery rate correction, *P* < 1E-05) (S1 Table). Among these genes, a relatively large number of genes related to stress response, especially ER stress, were identified by searching literature. For example, significant upregulation of HSR gene expression was observed including genes encoding the heat shock 70kDa protein 6 (HSP70B') (*HSPA6*; 19.93-fold), heat shock 70kDa protein 1A (*HSPA1A*; 2.45-fold), DnaJ (Hsp40) homolog, subfamily A, member 4 (*DNAJA4*; 2.01-fold), heat shock 70kDa protein 1B (*HSPA1B;* 1.90-fold), DnaJ (Hsp40) homolog, subfamily B, member 1 (*DNAJB1*; 1.64-fold) and heat shock 70kDa protein 8 (*HSPA8*; 1.62-fold). Moreover, upregulation of genes encoding activating transcription factor 3 (*ATF3*; 3.30-fold), the p53-regulated DNA damage inducible stress sensor growth arrest and DNA-damage-inducible, alpha (*GADD45A*; 1.88-fold), antioxidant enzyme heme oxygenase (decycling) 1 (*HMOX1*; 1.83-fold), and the ER stress responsive transcription factor DNA-damage-inducible transcript 3 (*CHOP/GADD153*; 1.70-fold) were detected as well (Table 1).

**Table 1:** PA upregulates expression of ER stress related genes.

Gene	Description	Fold change
***HSPA6***	Heat shock 70kDa protein 6 (HSP70B')	+19.93
***ATF3***	Activating transcription factor 3	+3.30
***HSPA1A***	Heat shock 70kDa protein 1A	+2.45
***DNAJA4***	DnaJ (Hsp40) homolog, subfamily A, member 4	+2.01
***HSPA1B***	Heat shock 70kDa protein 1B	+1.90
***GADD45A***	Growth arrest and DNA-damage-inducible, alpha	+1.88
***HMOX1***	Heme oxygenase (decycling) 1	+1.83
***CHOP***	DNA-damage-inducible transcript 3	+1.70
***DNAJB1***	DnaJ (Hsp40) homolog, subfamily B, member 1	+1.64
***HSPA8***	Heat shock 70kDa protein 8	+1.62

Expressions of different genes were detected by the Human Prime View Array technology in MIA PaCa-2 cells after 24 hours PA treatment with PA (0 and 30 μM) in quadruplicates. Gene expression levels were normalized and analyzed using Microarray Data Portal (MDP) by 2-way ANOVA using cell line and treatment as factors plus the interaction of cell line and treatment. A q-value program from Storey and Tibshirani was used to calculate false discovery rates. Fold change P < 1E-05.

### PA induces expression of ER stress response proteins

**Fig 2:**
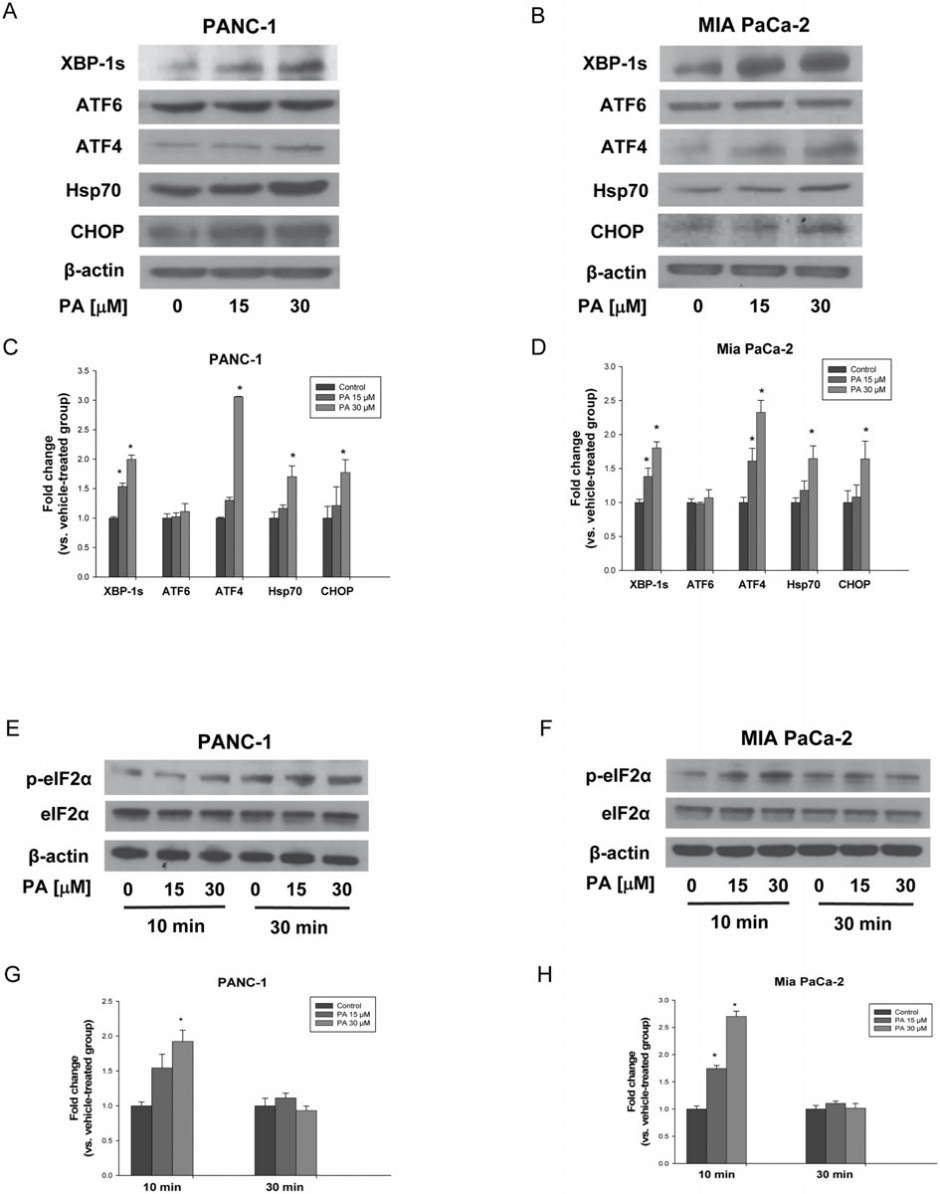
PA induces expression of ER stress response proteins. (A) PANC-1 and (B) MIA PaCa-2 cells were treated with PA (0–30 μM) for 24 h, respectively. Whole protein extracts isolated from cells were prepared and western blot analysis with anti-XBP-1s, anti-ATF6, anti-ATF4, anti-Hsp70 and anti-CHOP antibodies were performed as described in Materials and methods. (E) PANC-1 and (F) MIA PaCa-2 cells were treated with PA (0–30 μM) for 10 min and 30 min, respectively. Whole protein extracts isolated from cells were prepared and western blot analysis with anti-phospho-eIF2α and anti-eIF2α antibodies were performed as described in Materials and methods. β-actin was used as loading control. Representative blots from three experiments were shown and quantitative data composed of all the experiments in C), G) PANC-1 and D), H) MIA PaCa-2 cells with statistical analysis were below the representative blot image. *P<0.050 was considered to be significant when compared to control (n = 3) by Student t-test. The graphical data represent mean +/- SD.

We further evaluated if PA upregulates expression of ER stress related genes at the protein level. PANC-1 and MIA PaCa-2 cells were treated with PA (0–30 μM) for 10 min, 30 min or 24 h, respectively. Consistent with gene expression array data obtained at the mRNA level after 24 h treatment, induction of ER stress response in PANC-1 and MIA PaCa-2 cells were detected by western blot analysis of Hsp70 and CHOP. Moreover, two transcription factors involved in UPR system, XBP1and ATF4, were significantly activated as well (Fig 2A–2D).

In addition, the phosphorylated form of eIF2α (p-eIF2α), a sensitive indicator of ER stress and key regulator of UPR [7, 41], was detectable after 10 min PA treatment (30 μM) in PANC-1 and MIA PaCa-2 cells (Fig 2E and 2F). Our results showed that PA-phosphorylated eIF2α was nearly 2 fold in PANC-1 cells and 3 fold in MIA PaCa-2 cells comparing with control (Fig 2G and 2H).

### Blocking ER stress inhibits PA induced apoptosis

**Fig 3:**
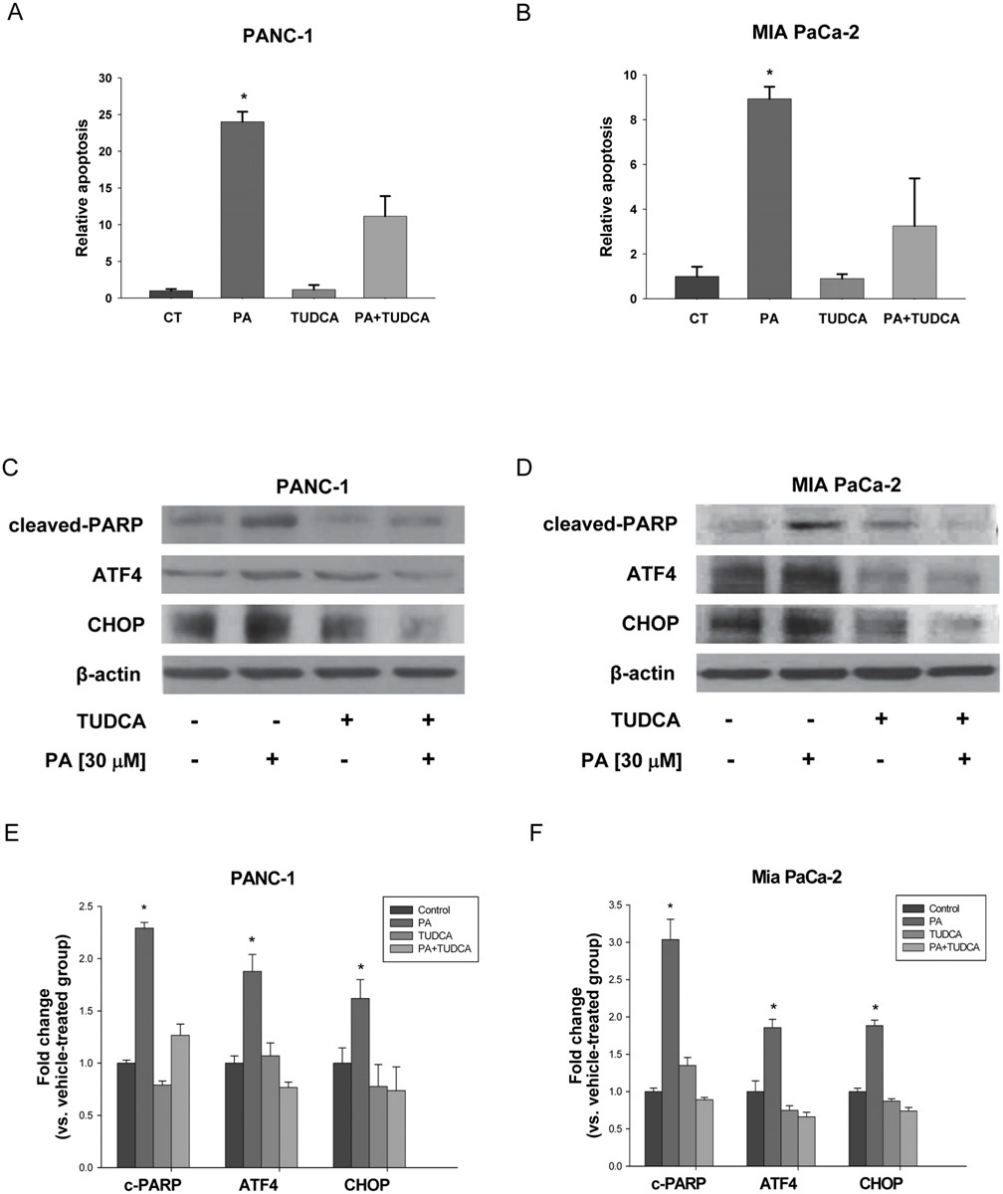
Blocking ER stress inhibits PA induced apoptosis. (A) PANC-1 and (B) MIA PaCa-2 cells were treated with or without PA (30 μM) in presence of TUDCA (200 μM) for 24 h. Apoptosis was detected by Cell Death Detection ELISA and expressed relative to vehicle-treated cells (set equal to 1). Data were compared by ANOVA with Bonferroni correction for the significant level. Here, *p≤0.025 was considered significant for the individual t-tests. Apoptosis was confirmed in C) PANC-1 and D) MIA PaCa-2 cells treated with same concentrations of PA in presence of TUDCA (200 μM) for 24 h. Representative blots show expression of PARP cleavage (c-PARP) and β-actin was used as loading control. Effect of TUDCA on PA induced expressions of ATF4 and CHOP in (C) PANC-1 and (D) MIA PaCa-2 cells were shown as well. Three independent experiments were done for the western blot studies and quantitative data composed of all the experiments in E) PANC-1 and F) MIA PaCa-2 cells with statistical analysis were shown below the representative blot image. *P<0.050 was considered to be significant when compared to control (n = 3) by Student t-test. The graphical data represent mean +/- SD.

As we observed significantly enhanced protein expression of XBP-1s, ATF4, Hsp70 and CHOP by PA treatment, it is necessary to confirm that ER stress is induced in our model. We used tauroursodeoxycholic acid (TUDCA), a known chemical chaperone that acts as an ER stress inhibitor to block ER stress [42–45]. As shown in Fig 3C and Fig 3D, TUDCA treatment blocked the induction of ATF4 and CHOP in both PANC-1 and MIA PaCa-2 cells. These results strongly support that PA activates ER stress in pancreatic cancer cells.

To establish that ER stress induced by PA leads to apoptotic cell death, we treated pancreatic cancer cells with PA plus TUDCA. Our results show that TUDCA treatment blocked the enormous increase in the expression of c-PARP associated with PA (Fig 3C–3F). In addition, the induction of nuclear DNA fragmentation associated with PA was rescued by TUDCA treatment (Fig 3A and 3B). These results support our hypothesis that ER stress was involved in the regulation of PA mediated apoptosis in pancreatic cancer cells.

### PA inhibits growth of chemoresistant pancreatic tumors

**Fig 4:**
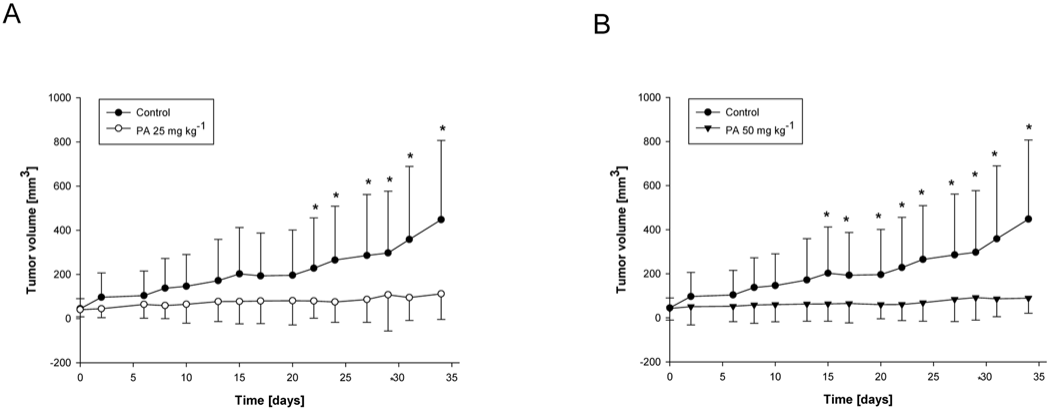
PA inhibits growth of pancreatic tumors *in vivo*. MIA PaCa-2 cells were subcutaneously implanted into female nude mice and treated with PA (A) 0 and 25 mg kg^-1^, and (B) 0 and 50 mg kg^-1^ of body weight 3 times week^-1^ as described in Materials and methods. Tumor sizes were measured by microcalipers 3 times per week and calculated for additional 5 weeks. Data are mean ± SD (n = 10–15). T-test was used to compare the mean tumor volumes of both treatment groups to control at day 34 (*p = 0.004).

To confirm our observation *in vivo*, we employed a xenograft model of chemotherapy resistant pancreatic cancer. A mixed effect general linear model was used to assess the change in tumor volume across groups over time and mice were included as a random effect. A compound symmetric covariance structure was assumed and interaction between groups and time in the model is of primary interest. There were significant differences in tumor volume between treatment groups and control group over time. 25 mg kg^-1^ and 50 mg kg^-1^ of PA both obviously suppressed the growth of chemotherapy resistant pancreatic tumors (Fig 4A and 4B). To further emphasize the difference between groups, t-test was used to compare the mean tumor volumes of treatment groups to control at day 34. Both tests had p = 0.004, showing that at day 34, the tumor volumes in each treatment group was significantly different from control. The average tumor volume of mice in control group was approximately 448 mm^3^ comparing with about 112 mm^3^ in 25 mg kg^-1^ or 89 mm^3^ in 50 mg kg^-1^ PA treatment group, which was nearly 75% or 80% inhibition, respectively (Fig 4A and 4B).

### PA induces apoptosis in pancreatic tumors

**Fig 5:**
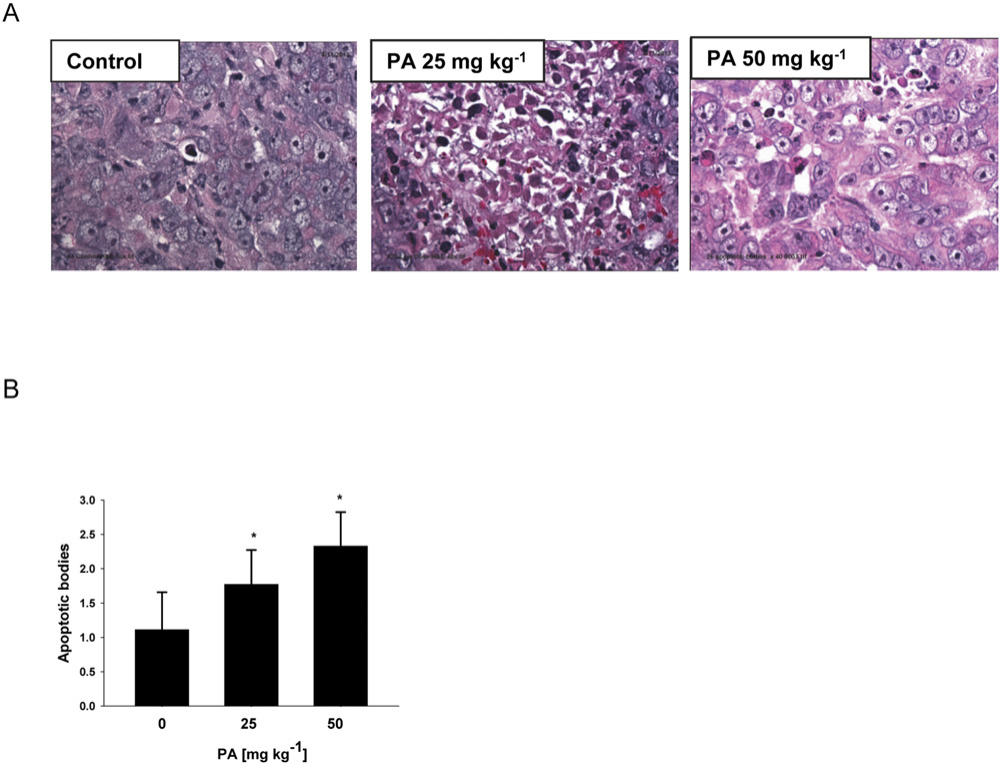
PA induces apoptosis in pancreatic tumors. Animal experiments were performed as described in Fig 4. (A) Representative H&E staining in pancreatic tumors, (B) quantification of apoptotic bodies was determined as described in Materials and methods. Data are mean ± SD (n = 6–12). Control, 25 mg PA kg^-1^ and 50 mg PA kg^-1^ were compared by ANOVA with Bonferroni correction for the significant level. Here, *p≤0.025 was considered significant for the individual t-tests.

Based on our convincing *in vitro* results, we hypothesized that PA inhibits growth of chemotherapy resistant pancreatic tumor *in vivo* through induction of apoptosis in tumor cells. Apoptosis is characterized by marked changes in cell morphology, including nuclear breakdown, chromatin condensation, and appearance of membrane-associated apoptotic bodies [46]. In order to prove our hypothesis, the amounts of apoptotic bodies in pancreatic tumor xenografts were quantified. H&E staining revealed significantly more brown apoptotic bodies in the tumor tissues from PA treated mice as compared with controls (Fig 5A), indicating that tumor growth inhibition was due to apoptosis induced by PA treatment. Quantification results showed approximately 59% increase of apoptotic bodies in 25 mg kg^-1^ group and 2 fold increase in 50 mg kg^-1^ group comparing with control (Fig 5B).

### PA (25 mg kg^-1^) is not toxic in xenograft model

**Fig 6:**
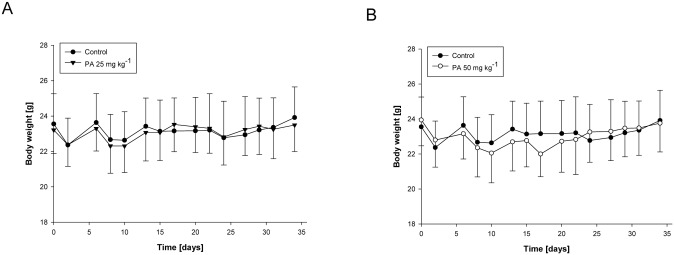
Effect of PA on the body weight of mice. Animal experiments were performed as described in Fig 4. Body weights were measured 3 times per week for (A) 0 and 25 mg kg-1, and (B) 0 and 50 mg kg−1 PA treatment. The graphical data represent mean +/- SD.

**Fig 7:**
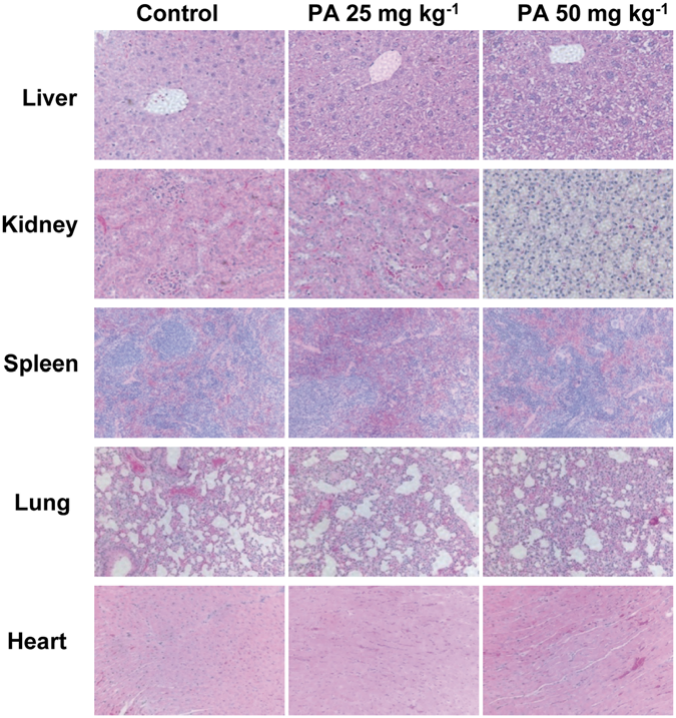
Evaluation of organ pathology with PA treatment. Animal experiments were performed as described in Fig 4. Liver, kidney, spleen, lung and heart tissues were stained with H&E. Images are representative of control and PA treatment (25 mg kg^-1^ and 50 mg kg^-1^) groups.

Although PA inhibits growth of chemotherapy resistant pancreatic tumors *in vivo*, its possible toxicity must be tested comprehensively. During the pancreatic tumor xenograft experiments, there were no significant differences in body weight between control and treatment groups (Fig 6A and 6B). However, 50 mg kg^-1^ of PA treated mice showed several signs of discomfort and impaired movement comparing with control and 25 mg kg^-1^ of PA treated mice. It suggested that 50 mg kg^-1^ of PA treatment might be toxic to mice. Moreover, H&E staining results showed that 25 mg kg^-1^ of PA treatment did not demonstrate any abnormalities in liver, kidney, spleen, lung and heart tissues but there were obvious pathological changes in liver, kidney and spleen tissues from 50 mg kg^-1^ of PA treated mice (Fig 7).

In addition, liver enzyme profiles in plasma including ALP, ALT, AST, albumin and total protein levels were not markedly altered in 25 mg kg^-1^ of PA treatment group. However, AST, albumin and total protein levels were significantly increased in 50 mg kg^-1^ of PA treated mice (Table 2). In conclusion, although 50 mg kg^-1^ of PA treatment has some toxic effect *in vivo*, no major toxicity was observed in tested animals from 25 mg kg^-1^ of PA treatment group.

**Table 2:** Effect of PA on liver enzyme profile of mice.

PA [mg kg^-1^]	ALP [IU L^-1^]	ALT [IU L^-1^]	AST [IU L^-1^]	Albumin [mg dL^-1^]	T Protein [mg dL^-1^]
**0**	60 ± 19	123 ± 88	625 ± 367	1.5 ± 0.2	4.1 ± 0.5
**25**	62 ± 9	194 ± 193	531 ± 296	1.6 ± 0.1	4.5 ± 0.2
**50**	59 ± 11	73 ± 736*	1431 ± 997*	1.6 ± 0.1	4.6 ± 0.4*

Animal experiments were performed as described in Fig 4. Values are Mean ± SD (n = 10–13). ALP, alkaline phosphatase; ALT, alanine aminotransferase; AST, aspartate aminotransferase; T protein, total protein. Blood was collected and serum was analyzed at the IU Health Pathology Laboratory.

*P < 0.050 for 50 mg kg^-1^ PA vs. control by ANOVA.

### PA induces ER stress in pancreatic tumors

**Fig 8:**
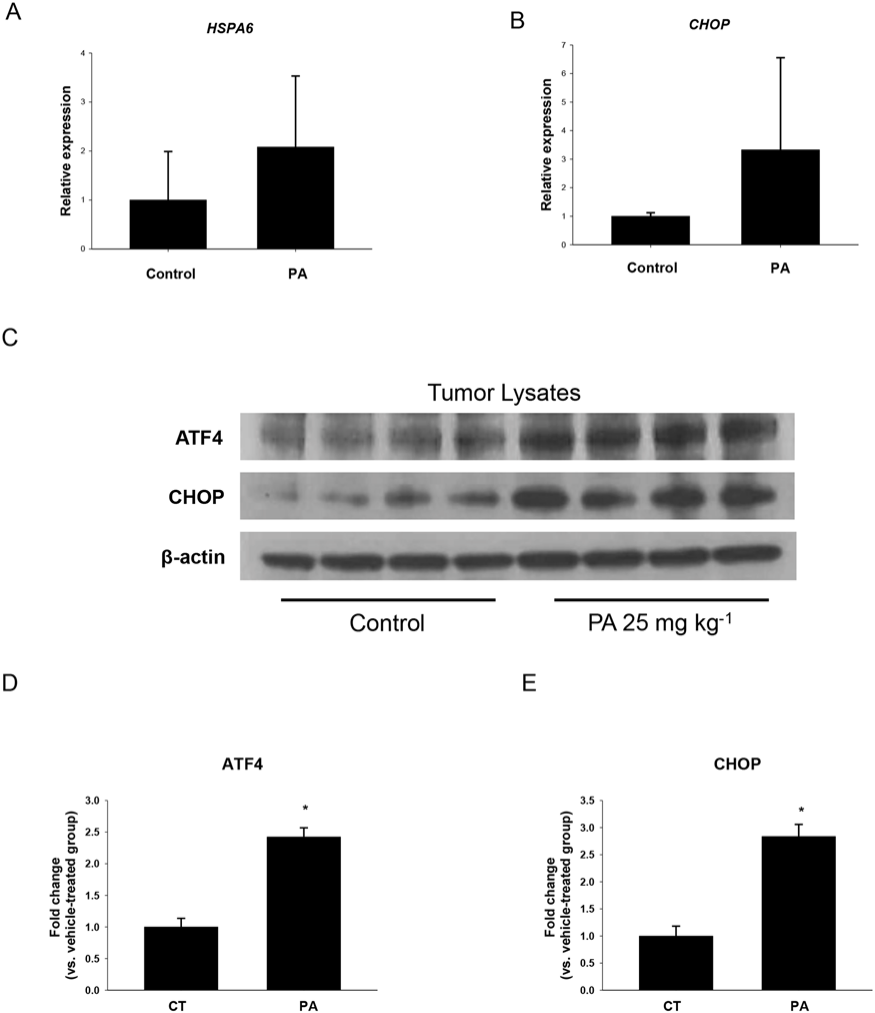
PA upregulates expression of ER stress related proteins *in vivo*. Animal experiments were performed as described in Fig 4. Expressions of (A) *HSPA6* and (B) *CHOP* in tumor tissues were determined by qRT-PCR and normalized to *ACTB* expression as described in Materials and methods. *P<0.050 was considered to be significant when compared to control (n = 8–10) by Student t-test. (C) Expressions of ATF4 and CHOP in tumor tissues were detected by western blot analysis. β-actin was used as loading control. Expressions of (D) ATF4 and (E) CHOP were quantified with statistical analysis. *P<0.050 was considered to be significant when compared to control (n = 7) by Student t-test. Data are expressed as the fold change *vs*. vehicle-treated cells (set equal to 1). The graphical data represent mean +/- SD.

To assess whether non-toxic dose of PA mediated tumor growth suppression was associated with induction of ER stress, RNA of tumors from control and 25 mg kg^-1^ of PA treated mice were isolated and subjected to quantitative RT-PCR. However, the significant increase of ER stress related genes, such as *HSPA6* which encoding the heat shock 70kDa protein 6 (HSP70B') and *CHOP/GADD153*, were not observed in the tumors of PA treated mice comparing with respective controls (Fig 8A and 8B). In addition, the expression of *HSPA1A*, which encoding the heat shock 70kDa protein 1A, was not detectable in each group (data not shown). In order to investigate the changes in the protein levels which are more convincing, tumors from control and 25 mg kg^-1^ of PA treated mice were lysed and analyzed by western blot. Interestingly, a significant increase in the expression of ER stress related proteins, such as ATF4 and CHOP, were shown in the tumors of PA treated mice as compared to controls (Fig 8C–8E). These *in vivo* observations are in accordance with our *in vitro* data based on MIA PaCa-2 cells. Our results indicate that non-toxic dose of PA suppresses the growth of chemotherapy resistant pancreatic tumor through inducing ER stress mediated apoptosis.

## Discussion and Conclusions

Pancreatic cancer responds poorly to current therapies, which make this malignancy especially challenging and a high priority for identification of novel effective drugs [17]. We previously demonstrated that PA behaves as the most effective compound in suppressing proliferation of human pancreatic cancer cells among the different structurally related triterpenes extracted from *P*. *cocos*, underlining the importance of molecular structure for their anti- cancer activities. Moreover, PA only slightly affects the growth of normal pancreatic duct epithelial cells HPDE-6 and also significantly suppresses invasive behavior of pancreatic cancer cells by inhibiting expression of MMP-7 [29]. The studies reported here for the first time indicating that PA is a potent anticancer agent against chemotherapy resistant pancreatic cancer *in vitro* and *in vivo*, and highlight the importance of ER as a target for the treatment of pancreatic cancer.

We observed that apoptosis induced by PA in chemotherapy resistant pancreatic cancer cells was not cell specific, as assessed in different gemcitabine-resistant pancreatic cancer cells PANC-1 and MIA PaCa-2 by detecting the presence of nuclear DNA fragmentation and PARP cleavage activation. Moreover, our data represent the first demonstration of the *in vivo* pro-apoptotic activity of PA, which further supports the notion that the remarkable antitumor action of PA highly depends on its ability to promote apoptosis in tumors. In addition, based on standard toxicology studies including body weight measurements, organ pathology and liver enzyme profile analysis, we confirmed that 25 mg kg^-1^ of PA treatment is not toxic *in vivo*. As recently demonstrated, PA was detectable in urine and plasma of rats fed by *P*. *cocos* [47], indicating that PA can be easily absorbed into blood. Therefore, the bioavailability of PA further supports development of PA in the treatment of pancreatic cancer.

Gene expression profiling by microarray has been widely used in screening drug targets [48]. In order to investigate the effect of PA and its possible mechanisms of action with regard to gemcitabine-resistant pancreatic cancer, we treated MIA PaCa-2 cells with PA and the expression of genes was monitored using array technology. Many cancer-related genes were significantly up and down-regulated upon PA treatment. Here, we report for the first time that the mechanisms modulating PA effect may be mediated through triggering a prolonged ER stress response in chemotherapy resistant pancreatic cancer cells, which leads to apoptosis.

**Fig 9:**
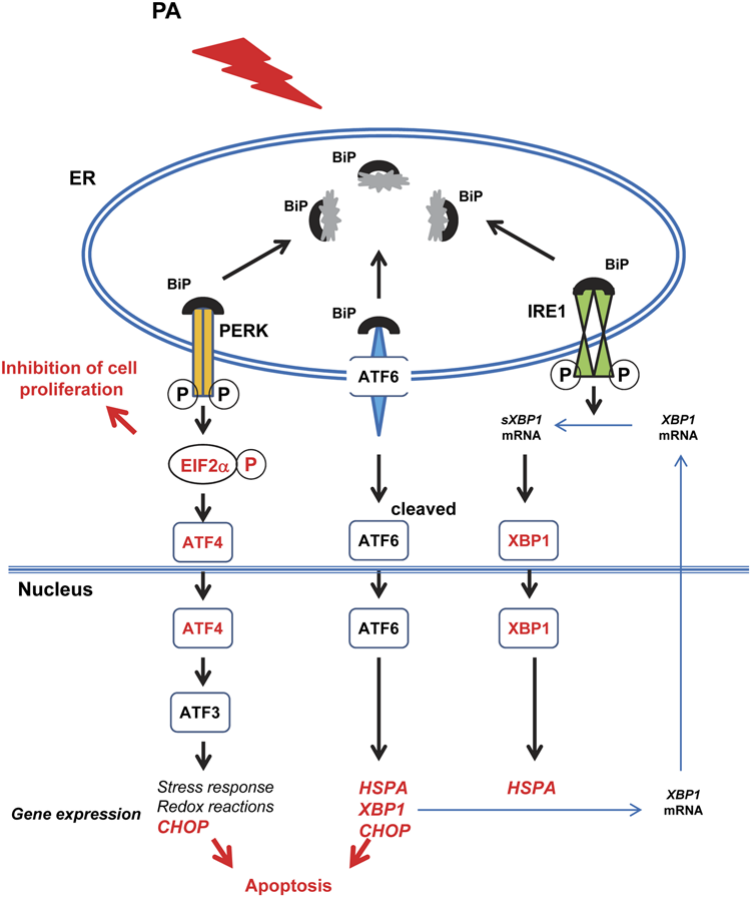
Model for the involvement of ER in PA-induced apoptosis and inhibition of tumor growth. Target molecules XBP-1s, ATF4, phospho-eIF2α, HSPA and CHOP are in red.

Fig 9 depicts a model for the involvement of ER in PA-induced apoptosis in chemotherapy resistant pancreatic cancer cells. The expression of HSR genes, such as *HSPA6*, *HSPA1A*, *DNAJA4*, *HSPA1B*, *DNAJB1* and *HSPA8*, were significantly upregulated to overcome the effects of ER stress [49]. Moreover, PA elevated the expression of *ATF3*. As a member of the ATF/CREB subfamily of the basic-region leucine zipper (bZIP) family [50], ATF3 works directly or indirectly as a transcriptional activator of genes targeted by the eIF2 kinase stress pathway in UPR, and phosphorylation of eIF2α by PERK is an early event in a coordinate gene expression response to ER stress [14, 51–53]. We found that PA promotes phosphorylation of eIF2α in pancreatic cancer cells PANC-1 and MIA PaCa-2 by western blot analysis, further supporting the idea that PA induced ER stress in chemotherapy resistant pancreatic cancer cells. Jiang et al. also reported that increased expression of ATF3 occurs early in response to stress through a mechanism requiring the related bZIP transcriptional regulator activating transcription factor-4 (ATF4) [54], which is in accordance with our data of western blot analysis *in vitro* and *in vivo*.

However, a severe and prolonged ER stress during exposure to PA leads to the induction of pro-apoptotic transcription factor CHOP/GADD153. CHOP, also known as DDIT3 (DNA-damage-inducible transcript 3), is involved in growth arrest and apoptosis following DNA damage and a variety of stress conditions, such as nutrient deprivation and treatment with anticancer agents [55]. It is induced by ATF3 in response to prolonged ER stress and is the downstream component of ATF4 [54, 56]. Increasing translation of ATF4 results in the transcriptional activation of CHOP [57]. Here, we found both *in vitro* and *in vivo* evidence for the up-regulation of ATF4-dependent protein CHOP following PA treatment in chemotherapy resistant pancreatic cancer cells, which is closely associated with the progression of apoptosis. Glucose-regulated protein 78 (GRP78), also called BiP, is one of the major regulators of ER stress which keeping the transcription of CHOP in a low level [44, 58, 59]. Recently, Nameeta et al. found the overexpression of GRP78 in the human pancreatic cancer cell lines and tissue samples. They also proved that GRP78 is downregulated by triptolide, which leading to apoptotic cell death of MIA PaCa-2 cells [60]. In the future studies, we will investigate if PA induces apoptosis of pancreatic cancer through inhibiting expression of the survival protein GRP78 as well.

There is published evidence that lanostane-type triterpenes possess antioxidant and free radical scavenging activities, which may play an important role in reducing cancer risk [61]. Ursolic acid, a pentacyclic triterpene acid widely distributed in apple peels, has both anti-apoptotic and antioxidative activities against ER stress-associated myocardial damage in mouse cardiac myocytes [62]. However, based on *in vitro* cell-based and *in vivo* mouse xenograft results in our study, we concluded that PA induced apoptosis of pancreatic cancer cells through activating ER stress. These seemingly contradicted results show that the activities of different triterpene acids are complicated, depending on the various structures, cell types, treatment times, concentrations and so on.

Conventional cancer treatments, such as inhibitors of mitosis or S phase, target all the proliferating cells, therefore cause many side effects [63]. Because of extrinsic factors (e.g. nutrient and oxygen deprivation) and intrinsic factors (e.g. high glucose metabolic rate) in the tumor microenvironment, cancer cells exhibit higher levels of ER stress compared to normal cells [64]. The stressed state of malignant cells makes them vulnerable to therapeutic strategies, which target molecular determinants of HSR or UPR or a combination of both to exacerbate enhanced stress in cancer selective therapy [10]. Recent studies showed that diindolylmethane (DIM) induces autophagy in ovarian cancer cells through regulating ER stress [65]. Our findings here indicate that PA exerts its pro-apoptotic action in chemotherapy resistant pancreatic cancer cells, both *in vitro* and *in vivo*, through induction of a sustained ER stress that eventually leads to apoptosis. Interestingly, PA only slightly affects the proliferation of normal pancreatic duct epithelial cells and effective dose of PA is not toxic *in vivo*, which is particularly important in tumor-selective therapy. These data suggest that ER targeting by PA may represent a promising new framework in the treatment of currently incurable pancreatic cancer. Further clinical studies are necessary to test if PA is well tolerated in humans and its effect in suppressing pancreatic tumor growth in humans.

## Supporting Information

S1 Table List of upregulated genes in pancreatic cancer cells with PA treatment. (XLSX)
